# Direct and indirect associations of hypochondriasis with suicidality in psychiatric outpatients: mediating roles of anxiety and depression

**DOI:** 10.3389/fpsyt.2026.1796129

**Published:** 2026-04-15

**Authors:** Joonyoung Lim, Haein Kim, Chaeyeon Yang, Hyeri Moon, Yunsu Kim, Jihye Ahn, Jihee Jang, Seog Ju Kim

**Affiliations:** Department of Psychiatry, Sungkyunkwan University College of Medicine, Samsung Medical Center, Seoul, Republic of Korea

**Keywords:** anxiety, depression, hypochondriasis, mediation, suicidality

## Abstract

**Introduction:**

Although both hypochondriasis and suicidality are common in psychiatric patients and related to anxiety and depression, their association in psychiatric patients remains unclear. This study investigated the direct association of hypochondriasis with suicidality and the indirect associations via anxiety and depression in psychiatric patients.

**Methods:**

Clinical records of 5484 psychiatric outpatients were reviewed. Hypochondriasis, Suicidality, Anxiety, and Depression were evaluated using the hypochondriasis item of the Hamilton Depression Rating Scale, the suicidality item of the 17-item Hamilton Depression Rating Scale (HAM-D_17_), the Hamilton Anxiety Rating Scale (HAM-A), and the 6-item subscale of the Hamilton Depression Rating Scale (HAM-D_6_), respectively. The associations among Hypochondriasis, Suicidality, Anxiety, and Depression were examined using a parallel mediation model. The model was estimated using the lavaan package in R with 10, 000 bootstrap resamples, adjusted for age and sex. Moderation by age and sex was also investigated.

**Results:**

Significant positive indirect associations via Anxiety (point estimate = 0.05, 95% CI [0.03, 0.06]) and Depression (point estimate = 0.17, 95% CI [0.15, 0.19]) were observed between Hypochondriasis and Suicidality. Conversely, the direct association between Hypochondriasis and Suicidality was also significant but in a negative direction (B = −0.16, p <.001). As the total indirect association was stronger than the direct association, the total association of Hypochondriasis with Suicidality was significantly positive (B = 0.05, p = 0.002). The negative direct association of Hypochondriasis with Suicidality was significantly stronger in younger patients (interaction term = 0.004, p <.001).

**Conclusion:**

Anxiety and depression mediated the association between hypochondriasis and increased suicidality. In contrast, hypochondriasis was associated with decreased suicidality after accounting for the mediators. As the indirect association was stronger than the direct association, hypochondriasis was associated with increased suicidality overall. The direct association between hypochondriasis and decreased suicidality was stronger in younger patients.

## 1 Introduction

Suicide is a major cause of death worldwide, as more than 700, 000 people die by suicide annually (1). This issue is particularly important among psychiatric patients whose suicidal risk has been reported to be seven times higher (2). Complicated pathways may exist from diverse psychiatric symptoms to suicidality in psychiatric patients.

Hypochondriasis, characterized by inappropriate preoccupation or fear regarding the possibility of a serious illness, is also a common symptom in psychiatric patients (3). Although the term “hypochondriasis” still represents both disease and symptom, the disease entity of hypochondriasis was renamed as illness anxiety disorder in the Diagnostic and Statistical Manual of Mental Disorders, Fifth Edition (DSM-5) (4). Hypochondriasis has been more widely recognized as a continuum of symptoms rather than a categorical entity (5).

Despite the high prevalence of hypochondriasis in psychiatric patients, the association between hypochondriasis and suicidality remains unclear (6), with one study reporting no significant association between hypochondriasis and suicidality (7), whereas other studies reported an association between hypochondriasis and decreased suicidality in patients with major depressive disorder (8, 9). Conversely, a recent cohort study reported increased suicidality among individuals with hypochondriasis (10). Interestingly, the same cohort study reported that the association between hypochondriasis and increased suicidality was substantially attenuated after adjusting for anxiety and depressive disorders, which commonly coexist with hypochondriasis (11). These data suggest that the association between hypochondriasis and suicidality depends on clinical contexts, especially coexistent anxiety and depression.

The inconsistent previous results on the association between hypochondriasis and suicidality may be explained by inconsistent mediation (12), where hypochondriasis may exert opposing effects on suicidality. Hypochondriasis may increase suicidality through the mediation of depression or anxiety, whereas it may decrease suicidality independently. Nevertheless, previous studies did not investigate in detail the direct and mediated indirect pathways from hypochondriasis to suicidality.

Therefore, the present study was conducted to explore the direct association of hypochondriasis with suicidality and its indirect association with suicidality via anxiety and depression in psychiatric outpatients. This study hypothesized that 1) hypochondriasis positively correlates with suicidality through the mediation of anxiety and depression (positive indirect associations) and 2) hypochondriasis negatively correlates with suicidality after accounting for anxiety and depression (negative direct association).

## 2 Materials and methods

### 2.1 Participants

The clinical records of 6197 outpatients (mean age 50.94 ± 19.82 years; 2379 men and 3818 women) who visited the Department of Psychiatry at Samsung Medical Center, Seoul, South Korea, from January 2018 to December 2022 were retrieved. After excluding 4 adolescent outpatients, 6, 193 adult outpatients remained. From this group, a total of 709 patients (mean age 71.86 ± 20.20 years; 254 men and 455 women) were excluded using listwise deletion due to missing or incomplete data on the Korean version of the 17-item Hamilton Depression Rating Scale (HAM-D_17_) (n = 707) and the Hamilton Anxiety Rating Scale (HAM-A) (n = 653), with 651 patients lacking valid scores on both scales. Finally, 5484 (mean age 48.26 ± 18.06 years; 2124 men and 3360 women) adults (aged ≥18 years) were included in this study. The study was approved by the Institutional Review Board of Samsung Medical Center (No. 2020–11-107), which granted an exemption from the requirement for written informed consent.

### 2.2 Measures

The HAM-D_17_ is one of the most widely used clinician-rated scales designed to evaluate the severity of depressive symptoms (13). It consists of 17 items rated by trained clinical psychologists or psychiatrists. The items are scored on either 3-point (0–2) or 5-point (0–4) scales. The HAM-A is also a widely used clinician-rated instrument designed to evaluate the severity of anxiety (14). It comprises 14 items, each rated on a 5-point scale (0–4) based on a clinical interview.

In the present study, Hypochondriasis was defined using the hypochondriasis item of HAM-D_17_ that measures hypochondriacal preoccupation and health-related concerns. The hypochondriasis item scores were defined as follows: 0 = not present; 1 = self-absorption with bodily functions; 2 = preoccupation with health; 3 = frequent complaints and requests for help; and 4 = hypochondriacal delusions. Although the validity of using the hypochondriasis item of HAM-D_17_ for measuring health anxiety has not been established, some previous studies suggest that this item might have possible meaningful relevance in certain clinical contexts (9, 15–17). Suicidality was also operationalized using the suicide item of HAM-D_17_, which measures suicidal ideation and attempts. The suicide item scores were defined as follows: 0 = absent; 1 = feels life is not worth living; 2 = wishes to be dead or has thoughts of possible death to self; 3 = suicidal ideas or gestures; and 4 = suicide attempts. The validity of using the suicide item of HAM-D_17_ for measuring suicidality has been established in a previous study (18). There were previous studies that employed the suicide item of HAM-D_17_ as outcome variable (19–23). Depression was characterized using the 6-item Hamilton Depression Rating Scale (HAM-D_6_). This is a subscale consisting of six items from HAM-D_17_ (items 1, 2, 7, 8, 10, and 13), which was considered to capture depressive symptoms more effectively than the entire HAM-D_17_ (24, 25). Anxiety was characterized using the total HAM-A score.

### 2.3 Statistical analysis

Bivariate Pearson correlation analyses were conducted to examine the associations among study variables, and independent t-tests were performed to explore sex differences. Hypochondriasis and Suicidality were treated as continuous variables in the main analyses; treating ordinal variables with five or more response categories as continuous has been shown to produce comparable results (26). Harman’s single-factor test was conducted on the HAM-D_17_ items to examine common variance. Parallel mediation pathway analysis was used to test the hypothesized mediation model, which analyzed the indirect associations of Hypochondriasis with Suicidality via two parallel mediators, viz., 1) Anxiety and 2) Depression. All pathways in the main model were adjusted for participant age and sex as covariates. The two mediators (Anxiety and Depression) were allowed to correlate. Multicollinearity among variables was assessed using the variance inflation factor (VIF).

The direct association (c’) between Hypochondriasis and Suicidality, the specific indirect associations (Anxiety: a_1_ × b_1_; Depression: a_2_ × b_2_), and the total indirect association (a_1_ × b_1_ + a_2_ × b_2_) were examined. The total association was defined as the sum of the direct and total indirect associations. p <.05 was considered statistically significant for the total association, direct association, and pathways. Bootstrap 95% confidence intervals and bootstrap standard errors based on 10, 000 resamples were calculated to determine the statistical significance of indirect associations (27, 28). An indirect association was considered statistically significant if the 95% CI did not include zero. The hypothesized mediation model was fully saturated (df = 0), and therefore model-fit indices were not applicable.

Considering the significant associations in the correlation analysis, we investigated whether age or sex moderates the parallel mediation model. To evaluate the moderating effect of age (a continuous moderator), a moderated mediation analysis was conducted using interaction terms. Age was mean-centered to facilitate interpretation. The model tested for interactions between age and all pathways of the mediation model along with controlling for sex. The estimate of the moderated mediation quantified the change in the association per 1-year increase in age. Age was then divided into young (mean age − 1 SD), mean, and old (mean age + 1 SD) to examine the conditional association, and simple slope analysis was used to present the results. Multigroup pathway analysis was applied to examine the moderating effect of sex (a categorical moderator). The parallel mediation model was estimated simultaneously for both male and female groups, and the difference in the indirect and direct associations was formally tested between the two groups. This analysis controlled for age as a covariate. For all moderation analyses, 95% bootstrap confidence intervals were estimated with 10, 000 resamples. A conditional indirect association (for age) or a difference in indirect associations (for sex) was considered statistically significant if its 95% confidence interval did not include zero.

To test the robustness of the findings, a series of sensitivity analyses were conducted. First, the validity of variable measurements was examined. The mediation model excluding participants who scored at the ceiling of the Hypochondriasis (score 4 = hypochondriacal delusions) was evaluated to ensure that hypochondriacal delusion fall within the health anxiety construct. Additionally, given the conceptual overlap between Depression and Anxiety, the mediation model excluding psychic anxiety item from Depression score was evaluated. Second, the ordinal nature of Hypochondriasis and Suicidality scores (ranging 0-4) were assessed. Polychoric correlations were calculated between ordinal variables and polyserial correlations between ordinal and continuous variables. The WLSMV method was used to treat these as ordinal variables in the mediation model, and the statistical significance of indirect effects was evaluated using Monte Carlo simulation-based 95% confidence intervals (10, 000 draws). Third, as this study is a cross-sectional study, the reverse model that switched predictor and outcome (e.g., Suicidality → Anxiety/Depression → Hypochondriasis) was evaluated.

All statistical analyses were conducted using R (Version 4.4.2, R Core Team, 2025). The lavaan package (29) was used for structural equation modeling and parallel mediation analysis with bootstrap resampling (10, 000 iterations). The psych package (30) was used for reliability analysis and descriptive statistics. Data preprocessing and manipulation were performed using tidyverse packages (31).

## 3 Results

### 3.1 Demographic characteristics

The final sample included 5, 484 patients. The included group was significantly younger than the excluded group (t = 29.6, p <.001), with no significant difference in sex distribution (χ^2^ = 2.12, p = .15). The scores of Hypochondriasis and Suicidality of the final sample were 1.05 ± 0.87 and 0.84 ± 1.00, respectively. The full distribution of Hypochondriasis was as follows: 0 (n = 1, 608, 29.3%), 1 (n = 2, 322, 42.3%), 2 (n = 1, 247, 22.7%), 3 (n = 297, 5.4%), and 4 (n = 10, 0.2%). For Suicidality, the distribution was: 0 (n = 2, 732, 49.8%), 1 (n = 1, 349, 24.6%), 2 (n = 989, 18.0%), 3 (n = 362, 6.7%), and 4 (n = 52, 0.9%). The Anxiety score was 15.85 ± 7.06, whereas the Depression score was 6.70 ± 3.30. The sociodemographic and clinical characteristics of the study population are presented in **Table 1**. Regarding correlations, no significant correlation was found between Hypochondriasis and Suicidality (r = −0.02, p = .173). Hypochondriasis demonstrated significant positive correlations with Anxiety (r = 0.38, p <.001) and Depression (r = 0.22, p <.001). Suicidality was significantly associated with Anxiety (r = 0.40, p <.001) and Depression (r = 0.57, p <.001). A significant correlation was observed between Anxiety and Depression (r = 0.68, p <.001). Age demonstrated significant positive correlations with Hypochondriasis (r = 0.25, p <.001) but negative correlations with Anxiety (r = −0.03, p = .013), Depression (r = −0.17, p <.001) and Suicidality (r = −0.24, p <.001). Women exhibited higher levels of Hypochondriasis (t = 4.03, p <.001), Anxiety (t = 7.00, p <.001), Depression (t = 4.59, p <.001), and Suicidality (t = 4.41, p <.001) than men. Women were younger than men among subjects in the present study (t = −7.87, p <.001). VIF values were as follows: Hypochondriasis = 1.28, Anxiety = 2.08, Depression = 1.93, age = 1.15, and sex = 1.02, indicating no significant multicollinearity. In Harman’s single-factor test, the first factor accounted for 32.8% of the total variance, suggesting that a single method factor did not dominate the data (32).

**Table 1:** Sociodemographic and clinical characteristics of the study participants.

Variables	Total (N = 5484)
Age, M ± SD	48.26 ± 18.06
Sex, N (%)	
Men	2124 (38.7%)
Women	3360 (61.3%)
Hypochondriasis, M ± SD	1.05 ± 0.87
Anxiety, M ± SD	15.85 ± 7.06
Depression, M ± SD	6.70 ± 3.30
Suicidality, M ± SD	0.84 ± 1.00

N, number of participants; M, mean; SD, standard deviation.

### 3.2 Direct and indirect association of hypochondriasis with suicidality

**Figure 1:**
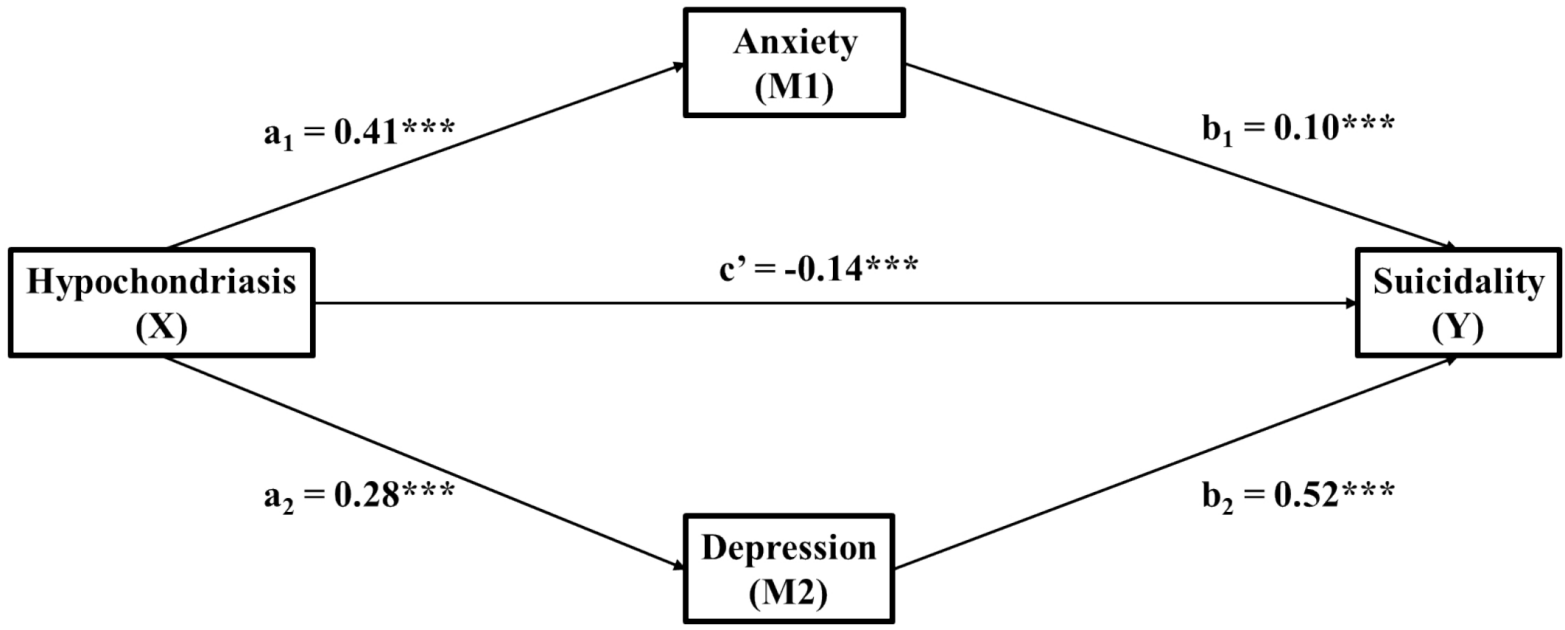
The parallel mediation model between Hypochondriasis and Suicidality via Anxiety and Depression. a_1_, Hypochondriasis → Anxiety; a_2_, Hypochondriasis → Depression; b_1_, Anxiety → Suicidality; b_2_, Depression → Suicidality; c', Direct association of Hypochondriasis → Suicidality. Standardized coefficients shown. All paths significant at ***p <.001. Model controlled for age and sex.

A parallel mediation model was applied to examine the association of Hypochondriasis with Suicidality via Anxiety and Depression controlling for age and sex, as shown in **Figure 1** and **Table 2**. Regarding Anxiety, Hypochondriasis correlated positively with Anxiety (pathway a1; B = 3.37, SE = 0.11, β = 0.41, p <.001), which in turn positively correlated with Suicidality (pathway b1; B = 0.01, SE = 0.002, β = 0.10, p <.001). Moreover, the indirect association via Anxiety was significantly positive (pathway a1 × b1; point estimate = 0.05, SE = 0.01, 95% CI [0.03, 0.06], fully standardized estimate = 0.04). This indicates that the association of Hypochondriasis with Suicidality was positively mediated by Anxiety.

**Table 2:** Direct and indirect associations of parallel mediation model linking hypochondriasis to suicidality via anxiety and depression.

Association	B	SE	95% CI	β	p
Direct Association	-0.16	0.02	[-0.19, -0.13]	-0.14	<.001
Total Indirect Association	0.21	0.01	[0.19, 0.24]	0.18	
Indirect via Anxiety	0.05	0.01	[0.03, 0.06]	0.04	
Indirect via Depression	0.17	0.01	[0.15, 0.19]	0.15	
Total Association	0.05	0.02	[0.02, 0.08]	0.04	.002

B, Unstandardized estimate; SE, Bootstrap standard error; 95% CI, Bootstrap 95% confidence interval; β, Fully standardized estimate. All associations were controlled for age and sex.

Regarding Depression, Hypochondriasis correlated positively with Depression (pathway a2; B = 1.07, SE = 0.05, β = 0.28, p <.001), which in turn positively correlated with Suicidality (pathway b2; B = 0.16, SE = 0.01, β = 0.52, p <.001). Moreover, the indirect association via Depression was significantly positive (pathway a2 × b2; point estimate = 0.17, SE = 0.01, 95% CI [0.15, 0.19], fully standardized estimate = 0.15). This indicates that the association of Hypochondriasis with Suicidality was positively mediated by Depression.

Since the specific indirect associations via Anxiety and Depression were positive, the total indirect association was also positive (pathway a1 × b1 + a2 × b2; point estimate = 0.21, SE = 0.01, 95% CI [0.19, 0.24], fully standardized estimate = 0.18). In other words, the positive association between Hypochondriasis and Suicidality is linked to elevated Anxiety and Depression.

In contrast to the indirect association, after controlling for both Anxiety and Depression, the direct association of Hypochondriasis with Suicidality was significantly negative (pathway c’; B = −0.16, SE = 0.02, β = −0.14, p <.001). This suggests that Hypochondriasis was associated with decreased Suicidality independent of the mediators. Since the direct association (β = −0.14) operated in the opposite direction to the indirect association (fully standardized estimate = 0.18) but was smaller in magnitude, the total association of Hypochondriasis with Suicidality was positive (pathway c; B = 0.05, SE = 0.02, β = 0.04, p = .002). Notably, this total association was significant only after adjusting for age and sex, as the unadjusted bivariate correlation was not significant (r = −0.02, p = .17).

### 3.3 Moderating effects of age and sex

**Figure 2:**
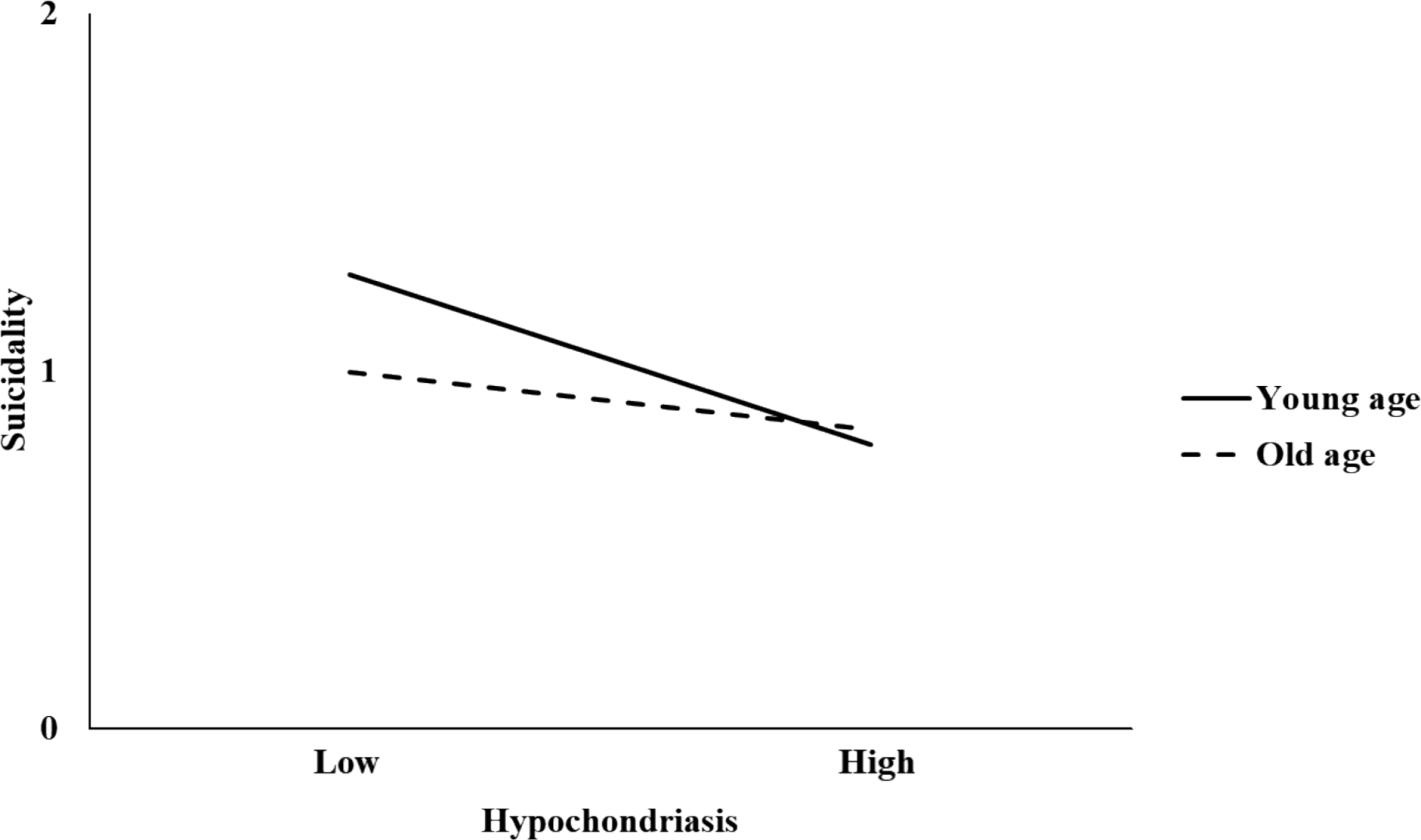
Moderation of the direct association of Hypochondriasis with Suicidality by age. Young age, mean age − 1 SD; Old age, mean age + 1 SD.

Age exerted a significant moderating effect on the following two pathways: 1) the association between Depression and Suicidality where age exhibited a negative interaction (interaction term = −0.001, p <.001), implying that the association between Depression and Suicidality was stronger in younger patients, and 2) the direct association between Hypochondriasis and decreased Suicidality where age exhibited a positive interaction (interaction term = 0.004, p <.001), implying that the direct association between Hypochondriasis and decreased Suicidality was stronger in younger patients. Specifically, the direct association of Hypochondriasis with Suicidality was significant at young age (mean age − 1 SD = 30.20 years; B = −0.24, 95% CI [−0.29, −0.20]), mean age (48.26 years; B = −0.16, 95% CI [−0.19, −0.13]), and old age (mean age + 1 SD = 66.32 years; B = −0.09, 95% CI [−0.12, −0.04]). The conditional direct associations of Hypochondriasis with Suicidality at different ages are illustrated in **Figure 2**. The remaining pathways were not moderated by age (all p >.05). The indirect association of neither Anxiety nor Depression exhibited significant moderation.

Sex did not significantly moderate the parallel mediation pathways. All pathways remained significant in both men (n = 2124) and women (n = 3360); however, the multigroup analysis revealed no significant sex differences in the indirect associations via Anxiety and Depression. The direct association also did not differ according to sex (all p >.05).

### 3.4 Sensitivity analysis

The findings remained robust in sensitivity analyses (**Supplementary Materials**). First, when assessing the variable measurement by excluding the ceiling of Hypochondriasis score or the psychic anxiety item of Depression, the direction and significance of all associations among study variables were preserved. Second, the correlation and mediation were consistent with the main findings when treating Hypochondriasis and Suicidality as ordinal variables. Third, although the direction and significance of all associations were preserved in the reverse model, the explanatory power for the outcome variable was higher in the original model (R^2^ = 0.37) than reverse model (R^2^ = 0.24).

## 4 Discussion

This study explored the direct and indirect pathways between hypochondriasis and suicidality. As hypothesized, anxiety and depression mediated the indirect association between hypochondriasis and increased suicidality. Interestingly, after adjusting for anxiety and depression, hypochondriasis was associated with decreased suicidality. These findings support inconsistent mediation, in which the indirect and direct associations operate in opposite directions. As the magnitude of indirect association was larger than the direct association, hypochondriasis was associated with increased suicidality in the total (indirect + direct) association. Furthermore, the present study showed that the direct association between hypochondriasis and decreased suicidality was stronger in younger patients.

The finding that anxiety mediated the association between hypochondriasis and increased suicidality is consistent with previous studies that reported a positive association between hypochondriasis and anxiety (33) and anxiety as a risk factor for increased suicidality (34). Nevertheless, to the best of our knowledge, the mediating role of anxiety linking hypochondriasis and suicidality has not yet been investigated. Hence, this result suggests that the pathway of hypochondriasis increases suicidality through increased anxiety. The fear of catastrophic illness and hypervigilance, the core features of hypochondriasis (35, 36), could contribute to anxiety (37, 38). On the other hand, there could be possible confounding effects of anxiety sensitivity that are commonly related to hypochondriasis (36), anxiety (39), and suicidality (40). In addition, bidirectional relationships could exist wherein somatic symptoms of anxiety could be misinterpreted and contribute to hypochondriasis.

Depression also mediated the association between hypochondriasis and increased suicidality. Despite the common comorbidity of diagnosed hypochondriasis and depressive disorder (11), the association between hypochondriasis and depression at symptom-level remains unclear (33). The present study demonstrated a positive association between hypochondriasis and depression. Depression was closely associated with suicidality, consistent with previous research (41). To the best of our knowledge, the mediating role of depression linking hypochondriasis and suicidality also remains unexplored. Hence, the result of this study suggests the pathway from hypochondriasis to increased suicidality through increased depression. The negative perspective and preoccupation with one’s own health might contribute to negative views of self and future, which is the core cognitive bias of depression (42). Nevertheless, this study could not exclude the reciprocal effect that cognitive distortion of depression (e.g., selective abstraction and overgeneralization) might also contribute to hypochondriasis. Future longitudinal studies would help clarify the relationship among anxiety, depression, and hypochondriasis.

Interestingly, hypochondriasis was associated with decreased suicidality after eliminating the mediation of anxiety and depression. While speculative due to the lack of direct measurement, a plausible explanation for this negative direct association would be the theory regarding death anxiety. Death anxiety, or the fear of death, is considered a primordial construct underlying various human behaviors and mental distress (43, 44). Not surprisingly, death anxiety was closely associated with hypochondriasis and recognized as one of its core cognitive features (45–47). A recent study reported that death anxiety was associated with factors of decreased suicidality, such as decreased wish to die, low suicide intention, and less severe circumstances of suicide attempt (48). Individuals with a strong fear of death may abstain from suicide intention and commit less fatal attempts. Therefore, the death anxiety of hypochondriasis may account for the potential protective effect against suicide.

The potential protective effect of hypochondriasis on suicidality was stronger in younger patients. Death anxiety could also be a plausible explanation for the stronger association between hypochondriasis and decreased suicidality in younger patients. Consistent with this, previous studies have reported higher death anxiety among younger individuals (49, 50). Therefore, the higher death anxiety among younger patients might contribute to the stronger direct association between hypochondriasis and decreased suicidality. Conversely, there was no moderating effect of sex. Studies on the role of sex in death anxiety have reported inconsistent results, with some studies reporting higher death anxiety among females (49, 51), whereas others reported no significant difference (50, 52). Studies have also suggested that the role of sex in death anxiety was rather based on social gender role and culture than biological sex (53, 54).

To the best of our knowledge, this is the first study to explore the association between hypochondriasis and suicidality in psychiatric outpatients, and the findings may have clinical implications. To address the increased risk of suicide in psychiatric outpatients with hypochondriasis, coexisting anxiety and depression, which mediate the association between hypochondriasis and increased suicidality, might be potentially relevant clinical considerations.

This study has several limitations. First, due to the cross-sectional design, causal or temporal relationships could not be established. Further longitudinal, prospective studies could examine the causal relationship. Second, data were collected from a single hospital and psychiatric outpatients, and the excluded patients were significantly older than the included patients, which may limit the generalizability of findings. Third, the use of single HAM-D_17_ item for measuring hypochondriasis lacks established validity. The floor effect might constrain the interpretability of the findings as nearly half of participants scored zero on the suicide item and 30% on the hypochondriasis item. Further studies using more precise and well-established scales such as the Health Anxiety Inventory or the Beck Scale for Suicidal Ideation would yield more robust findings. Fourth, the current study adjusted only for age and sex as covariates. The omission of clinical factors linked to suicidality in psychiatric outpatients (e.g., diagnostic profile, treatment, medication, history of suicide attempts, substance use, personality and other medical illness) could lead to residual confounding. Fifth, the large sample size of the present study may have yielded statistical significance for associations that are small in magnitude, even when their clinical meaning is uncertain. Finally, although death anxiety plays a vital role in our discussion, it was not directly measured in this study. Future studies should measure death anxiety and use it as another mediator variable.

To summarize, this study reported opposing direct and indirect associations of hypochondriasis with suicidality in psychiatric outpatients. Anxiety and depression mediated an indirect association between hypochondriasis and increased suicidality. Interestingly, after accounting for anxiety and depression, hypochondriasis was associated with decreased suicidality. As the indirect association was stronger than the direct association, hypochondriasis was associated with increased suicidality overall. The direct association between hypochondriasis and decreased suicidality was stronger in younger patients.

## Data Availability

The raw data supporting the conclusions of this article will be made available by the authors, without undue reservation.
