# Supplementary material for: Direct and indirect associations of hypochondriasis with suicidality in psychiatric outpatients: mediating roles of anxiety and depression
Source: Front Psychiatry. 2026 Apr 15;17:1796129. doi: 10.3389/fpsyt.2026.1796129 (PMC13125137; doi:10.3389/fpsyt.2026.1796129)
Supplement: Supplementary file 1 [file Table1.docx]

**Supplementary Table 1.** Direct and indirect associations of parallel mediation model linking Hypochondriasis to Suicidality via Anxiety and Depression, excluding the ceiling score of the Hypochondriasis (score 4 = hypochondriacal delusions)

| Association | B | SE | 95% CI | β | p |
| --- | --- | --- | --- | --- | --- |
| Direct Association | -0.17 | 0.02 | [-0.19, -0.14] | -0.14 | <.001 |
| Total Indirect Association | 0.22 | 0.01 | [0.20, 0.24] | 0.19 |  |
| Indirect via Anxiety | 0.05 | 0.01 | [0.03, 0.06] | 0.04 |  |
| Indirect via Depression | 0.17 | 0.01 | [0.15, 0.19] | 0.15 |  |
| Total Association | 0.05 | 0.02 | [0.02, 0.08] | 0.04 | .001 |

Note. B = Unstandardized estimate; SE = Bootstrap standard error; 95% CI = Bootstrap 95% confidence interval; β = Fully standardized estimate. All associations were controlled for age and sex.

**Supplementary Table 2.** Direct and indirect associations of parallel mediation model linking Hypochondriasis to Suicidality via Anxiety and Depression, excluding psychic anxiety item from Depression score

| Association | B | SE | 95% CI | β | p |
| --- | --- | --- | --- | --- | --- |
| Direct Association | -0.15 | 0.01 | [-0.17, -0.12] | -0.13 | <.001 |
| Total Indirect Association | 0.19 | 0.01 | [0.17, 0.22] | 0.17 |  |
| Indirect via Anxiety | 0.06 | 0.01 | [0.05, 0.08] | 0.06 |  |
| Indirect via Depression | 0.13 | 0.01 | [0.11, 0.15] | 0.11 |  |
| Total Association | 0.05 | 0.02 | [0.02, 0.08] | 0.04 | .002 |

Note. B = Unstandardized estimate; SE = Bootstrap standard error; 95% CI = Bootstrap 95% confidence interval; β = Fully standardized estimate. All associations were controlled for age and sex.

**Supplementary Table 3.** Polychoric, polyserial, and Pearson correlation coefficients among Hypochondriasis, Anxiety, Depression, Suicidality, and age

| Variables | Hypochondriasis | Anxiety | Depression | Suicidality | Age |
| --- | --- | --- | --- | --- | --- |
| Hypochondriasis | - |  |  |  |  |
| Anxiety | 0.41*** | - |  |  |  |
| Depression | 0.24*** | 0.68*** | - |  |  |
| Suicidality | -0.01 | 0.45*** | 0.64*** | - |  |
| Age | 0.27*** | -0.03* | -0.17*** | -0.27*** | - |

*** p < .001, ** p < .01, * p < .05
**Note:** Polychoric correlations were calculated between ordinal variables (Hypochondriasis and Suicidality), polyserial correlations between ordinal and continuous variables, and Pearson correlations among continuous variables (Anxiety, Depression, and age).

**Supplementary Table 4.** Direct and indirect associations of the parallel mediation model linking Hypochondriasis to Suicidality via Anxiety and Depression, accounting for ordinal variables using WLSMV estimation

| Association | B | SE | 95% CI | β | p |
| --- | --- | --- | --- | --- | --- |
| Direct Association | -0.17 | 0.02 | [-0.20, -0.14] | -0.16 | <.001 |
| Total Indirect Association | 0.23 | 0.01 | [0.21, 0.25] | 0.22 |  |
| Indirect via Anxiety | 0.05 | 0.01 | [0.04, 0.07] | 0.05 |  |
| Indirect via Depression | 0.18 | 0.01 | [0.16, 0.20] | 0.17 |  |
| Total Association | 0.07 | 0.02 | [0.03, 0.10] | 0.06 | <0.001 |

Note. B = Unstandardized estimate; SE = Standard error; 95% CI = Monte Carlo 95% confidence interval; β = Fully standardized estimate. Hypochondriasis and Suicidality were treated as ordinal variables. All associations were controlled for age and sex.

**Supplementary Table 5.** Direct and indirect associations of reverse parallel mediation model linking Suicidality to Hypochondriasis via Anxiety and Depression

| Association | B | SE | 95% CI | β | p |
| --- | --- | --- | --- | --- | --- |
| Direct Association | -0.15 | 0.01 | [-0.17, -0.12] | -0.17 | <.001 |
| Total Indirect Association | 0.18 | 0.01 | [0.17, 0.20] | 0.21 |  |
| Indirect via Anxiety | 0.14 | 0.01 | [0.12, 0.15] | 0.16 |  |
| Indirect via Depression | 0.05 | 0.01 | [0.03, 0.07] | 0.05 |  |
| Total Association | 0.04 | 0.01 | [0.01, 0.06] | 0.04 | .002 |

Note. B = Unstandardized estimate; SE = Bootstrap standard error; 95% CI = Bootstrap 95% confidence interval; β = Fully standardized estimate. All associations were controlled for age and sex.
